# Use of the Bayley Scales of Infant and Toddler Development, Third Edition, to Assess Developmental Outcome in Infants and Young Children in an Urban Setting in South Africa

**DOI:** 10.1155/2017/1631760

**Published:** 2017-08-01

**Authors:** Daynia E. Ballot, Tanusha Ramdin, David Rakotsoane, Faustine Agaba, Victor A. Davies, Tobias Chirwa, Peter A. Cooper

**Affiliations:** ^1^Division of Neonatology, Department of Paediatrics and Child Health, University of the Witwatersrand, Private Bag X 39, Johannesburg 2000, South Africa; ^2^Division of Epidemiology and Biostatistics, School of Public Health, University of the Witwatersrand, Johannesburg, South Africa

## Abstract

**Background:**

The Bayley Scales of Infant and Toddler Development (III) is a tool developed in a Western setting.

**Aim:**

To evaluate the development of a group of inner city children in South Africa with no neonatal risk factors using the Bayley Scales of Infant and Toddler Development (III), to determine an appropriate cut-off to define developmental delay, and to establish variation in scores done in the same children before and after one year of age.

**Methods:**

Cohort follow-up study.

**Results:**

74 children had at least one Bayley III assessment at a mean age of 19.4 months (95% CI 18.4 to 20.4). The mean composite cognitive score was 92.2 (95% CI 89.4 to 95.0), the mean composite language score was 94.8 (95% CI 92.5 to 97.1), and mean composite motor score was 98.8 (95% CI 96.8 to 101.0). No child had developmental delay using a cut-off score of 70. In paired assessments above and below one year of age, the cognitive score remained unchanged, the language score decreased significantly (*p* = 0.001), and motor score increased significantly (*p* = 0.004) between the two ages.

**Conclusion:**

The Bayley Scales of Infant and Toddler Development (III) is a suitable tool for assessing development in urban children in southern Africa.

## 1. Background

Developmental assessment of infants is a difficult and time-consuming task. Standardized tools such as the Bayley Scales of Infant and Toddler Development provide a way of assessing a young child's development and comparing this to a standardized norm [1]. The Bayley Scales of Infant and Toddler Development, third edition (Bayley III), was published in 2006 and is a well-accepted tool that assesses development of children between the ages of 1 month and 42 months [1]. The Bayley III is an updated version of the Bayley Scales of Infant and Toddler Development, second edition (Bayley II). The Bayley II reports two scores of the mental development index (MDI) and the psychomotor index (PDI) [2]. In the Bayley III, cognitive development, expressive and receptive language, and fine and gross motor development are all evaluated. Composite scores are derived for cognitive, language, and motor development and scaled to a metric, with a mean of 100, standard deviation of 15, and range of 40 to 160. Results can also be expressed as percentile ranks relative to the standardization sample, with a mean and median of 50 and range from 1 to 99 [1]. The Bayley III normative population was a Western population whose mother tongue was English.

Developmental assessment of young children in low and middle income countries (LMICS) is a challenge due to socioeconomic, cultural, and language differences in the populations being tested. The Bayley III has been used to assess developmental outcome of children in South Africa; however there is very little published data on the performance of normal children. Much of the work done using the Bayley III in southern Africa was in assessing the development of HIV infected children where the group of HIV exposed but uninfected (HEU) was used as a control [3, 4]. Springer et al. have recently published a small study suggesting that there is no difference in neurodevelopmental outcome between HEU children and HIV unexposed uninfected children [5]. There is, however, increasing evidence that HEU children are a vulnerable group themselves [6]. Rademeyer and Jacklin published a study evaluating the use of the Bayley III in infants in South Africa [7]. Although this study investigated an important question, there were several important deficiencies; the sample size was small, half the infants were under the age of six months, and the results were compared to the normative Western Bayley population. Another study using the Bayley III showed that the developmental outcome of very low birthweight (VLBW) children in South Africa had developmental outcome within the normal range; however this study did not have a control group [8].

African children from a low socioeconomic background may perform poorly on the Bayley III for several reasons. There may be lack of an enabling environment at home due to a poor maternal level of education and poverty; poor children are not routinely exposed to educational toys and books which form a major part of the Bayley III tool. South Africa has eleven official languages; many children are exposed to more than one language at home. Some researchers have developed their own assessment tools to try and compensate for these differences, for example, the Malawi Development Assessment Tool [9]. Unfortunately, there are large differences between populations of children within Africa, including discrepancies in geographic location, socioeconomic status, and home language. It is therefore likely that an African based developmental assessment tool will be confounded by similar problems to the Bayley III.

Another question regarding the Bayley III relates to the most appropriate cut-off for defining developmental delay. A composite score of 70 seems to be too low and there are reports from studies using the Bayley III in high income countries which raise the concern that the Bayley III underestimates the number of children with developmental delay in comparison to Bayley II [2, 10]. A cut-off of 85 on the composite scores may be more appropriate in the Bayley III.

Loss to follow-up is a big problem in long-term follow-up studies in LMICS, including Africa. It is also much quicker to do a Bayley III assessment in an infant under the age of one year than in an older child. The question therefore arises as to whether the findings on a Bayley III assessment done under the age of one year remain the same at an older age. Springer et al. report a 67% follow-up rate after one year for infants recruited at birth [5]. An important question is whether early developmental assessment during infancy is sufficiently reliable to identify children with handicap. Although this question did not form part of their formal research, Rademeyer and Jacklin made the observation that children tested between two and six months of age tended to have higher scores on the Bayley III than those tested between seven and twelve months [7].

Many of the concerns outlined above can be addressed by the use of a control group from the same population when doing neurodevelopmental assessment studies in African settings. The control group provides a benchmark for comparison for the group of interest and shows the performance of normal children using a developmental assessment tool, such as the Bayley III.

The current study aims to describe the performance of a group of normal inner city children in South Africa using the Bayley III as compared to the Bayley normative population. Secondary objectives include evaluation of a cut-off of 70 or 85 to define handicap and to determine the variability in Bayley III scores done at different ages in the same child.

## 2. Methods

A group of well term neonates born between July 2013 and October 2013 who were discharged home with their mothers within 48 hours of birth was recruited. Children with obvious abnormalities, such as trisomy 21, likely to affect neurodevelopment were excluded. Mothers who had delivered in hospital were informed of the follow-up study and invited to attend a research newborn follow-up clinic. Children were enrolled into the follow-up study at the first newborn follow-up visit. Children were seen at three monthly intervals. Owing to an anticipated high study fallout rate after 1 year of age, Bayley III assessments were done at 9 to 12 months of age and again at 15 to 20 months of age. If a child defaulted, the mother was contacted via mobile telephone and rebooked at the clinic; the Bayley III was done at the next follow-up visit that was attended, regardless of age.

The Bayley III assessments were done by an appropriately trained physiotherapist or paediatrician. Cronbach's alpha intraclass correlation between different observers was 0.89. Intertest variation was evaluated comparing assessments done before and after twelve months of age in the same patients.

Measures taken to ensure a reasonable rate of follow-up included Short Message Service appointment reminders, refund of transport costs, tracing, and rebooking of defaulters. Children with developmental problems identified during the developmental assessment were referred to the appropriate paediatric or allied medical unit for therapy.

### 2.1. Statistical Analysis

Data were entered into a neonatal database, using Research Electronic Data Capture (REDCAP) software, hosted by the University of the Witwatersrand [11]. Data was exported into IBM SPSS 23 for statistical analysis. The Bayley III results were reported as the composite cognitive, language, and motor scores. All data were continuous variables with a normal distribution, so data were described using mean and 95% confidence intervals (95% CI). For the purposes of the primary aim, in children who had more than one assessment, the latest Bayley III assessment results were used. Paired *t*-tests were then used to compare the first and second Bayley III assessments done on the same children at different ages. Significance was considered at a level of 0.05.

Developmental delay was classified “at risk” if a Bayley III score was below 85 on any of the language, cognitive, or motor scales and as a “delayed” if a Bayley III score was below 70 on any of the subscales [8]. The mean (standard deviation) of the original normative Bayley population was 100 [1]. The original normative population data had a bell-shaped distribution; hence 15.86% of the original population had scores below 85 and 2.27% of the original population had scores below 70.

Sociodemographic factors were compared between children with normal versus delayed development. There were small numbers in this subanalysis, so categorical variables were compared using Chi square analysis and continuous variables using the Mann–Whitney *U* test.

### 2.2. Ethics

The study was approved by the Human Research Ethics Committee of the University of the Witwatersrand. Written informed consent was obtained from each parent prior to the child's enrolment in the study.

## 3. Results

**Figure 1:**
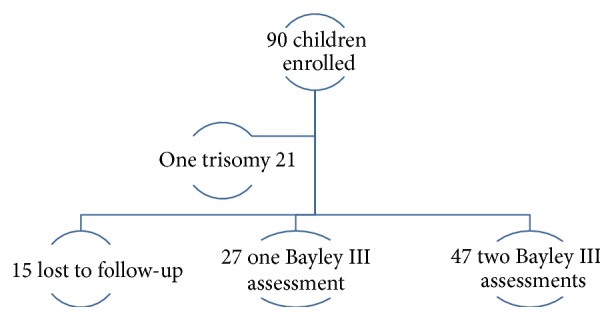
Enrolment of children for Bayley III assessment.

A total of 90 control children were enrolled for the follow-up study. One infant was diagnosed with trisomy 21 at the second follow-up visit and was excluded from the study. Of the remaining 89 children, 15 were lost to follow-up and 74 had at least one Bayley III assessment done giving a follow-up rate of 83.14%. There was a high drop-out rate at the 9- to 12-month visit; 27 (36.4%) children were successfully traced and rebooked. These 27 children only had one Bayley III assessment done after 12 months of age. The remaining 47 children (63.5%) had two Bayley III assessments (see Figure 1).

The mean birth weight of the children was 2674 grams (95% CI 2555–2803 grams). The mean maternal age was 27.67 years (95% CI 24.55–30.72 years). Gender distribution was equal between male and female. The majority (58/74 78.37%) of children were born by vaginal delivery. Almost one-third of the children were HIV exposed (22/74 29.72%), but all HIV PCR tests were negative. There were nine children (12.1%) who had an intercurrent hospital admission at some time during the follow-up. More than one-third of the mothers (26/74 35.13%) were not South African but came from surrounding African countries. Other sociodemographic information was available on 65/74 (87.8%) children (see Table 1).

A total of 74 children had at least one Bayley III assessment at a mean age of 19.4 months (95% CI 18.4–20.4). The mean cognitive score was 92.2 (95% CI 89.4–95.0), mean language score was 94.8 (95% CI 92.5–97.1), and mean motor score was 98.8 (95% CI 96.8–101.0). At the time of Bayley III assessment, the mean *Z* score for weight was 0.02 (95% CI −0.2 to 0.2), the mean *Z* score for height was −0.5 (95% CI −0.7 to −0.3), and the mean *Z* score for skull circumference was 0.5 (95% CI 0.2 to 0.7).

Two Bayley III assessments were done in the same 47 children at different ages. The results of these paired Bayley III assessments are compared in Table 2. The cognitive score remained unchanged, while the language score decreased and the motor score increased.

### 3.1. Developmental Delay

No children had a composite score below 70 for any of the subscales tested on any of the Bayley III assessments. Using a cut-off of 85, however, 19/74 (25.6%) of children had cognitive delay, which was approximately 1.6 times the normative data. There were 12/74 (16.2%) with language delay, which was more or less the same as the normative data. There were 4/74 (5.2%) with motor delay, which was less than expected from the normative data.

There was no difference in the number of children with a composite score below 85 in the first and second assessments in the 47 children with paired Bayley III tests (see Table 3).

There was no statistically significant difference in the *Z* score for weight, height, or skull circumference between children with normal and delayed development. Motor, cognitive, and language delay was not associated with gender, maternal age, maternal level of education, maternal employment, or place of abode.

## 4. Discussion

Developmental testing of infants and young children is a difficult and time-consuming task. The child's performance can be affected by factors such as intercurrent illness, a distracting environment, and the assessment tool used. The question also arises as to whether a Western tool such as the Bayley III is appropriate to use in an African setting. The Bayley III has been used in developmental outcome studies in southern Africa, frequently in the context of developmental assessment of children with HIV [4–7]; most of these Bayley III assessments were done in infants below the age of 1 year.

The current study showed that a group of inner city South African children without neonatal risk factors had scores within the normal range for cognitive, language, and motor function on the Bayley III assessment. The mean composite scores for each subscale were, however, all below 100 which is the 50th centile for the Bayley reference population [1]. The Bayley III scores in the current study are lower than those reported in other studies conducted in infants and young children in South Africa [4, 7]. This may be related to the older age of assessment in the present study. Rademeyer and Jacklin noted that the composite scores of children decreased with advancing age of assessment [7]. These findings suggest that developmental assessment of infants (i.e., below one year of age) may not be that reliable. It is better to do developmental assessment at an older age where possible. These findings highlight the importance of using a control group from the same population and of the same age when reporting developmental outcomes for research purposes.

There was a significant decrease in the language score in the present study. Below the age of 12 months, language development is assessed by determining generic response to noisemakers, babbling, and so on, while after 12 months, the tasks are much more specific; for example, child must identify objects in a picture book. Although this was not formally evaluated in the study, it is probable that most of these children are not exposed to books. Another problem with language assessment in the current study is that most children did not speak English as a home language. The assessor and child therefore interact through the mother who interprets the request. Translating the whole Bayley assessment into the vernacular would not solve this problem, as there are 11 official languages in South Africa. In addition, more than one-third of children in the current study were from other African countries and were thus exposed to additional different languages. The use of a translator during the testing could solve some of these issues.

Some of the tasks on both the cognitive and language scales may not be familiar to African children from a lower socioeconomic background. The level of difficulty of tasks in the Bayley III assessment increases as the normative age increases. Furthermore, several of the tasks on the cognitive scale in the Bayley III are incremental versions of the same activity, for example, finding a hidden object under a wash cloth. Thus, if the child fails to grasp the simplest form of the task, they will not be able to complete the more complex versions. It is possible that the child's lack of familiarity with the tasks may account for the relatively low cognitive score in the present study.

There is some discussion about the most appropriate cut-off for defining developmental delay in the Bayley III. Recent reports have suggested that a composite score of 70 on the Bayley III significantly underestimates the proportion of children with developmental delay [2, 8, 10]. In the current study, no child would be considered delayed using a cut-off score of 70. However, if a score of 85 was used to define delay, 25.6% of children would have cognitive delay, 16.2% language delay, and 5.2% motor delay. When compared to the original normative data, there were 1.6 times the number of children with cognitive scores <85, the proportion of children with language scores <85 was about the same, and there were fewer children with motor scores <85. A cut-off of 70 would therefore seem more appropriate to define developmental delay in the study setting. Children with a score below 85 could also be reported to be “at risk” of developmental delay [6].

## 5. Conclusion

The present study shows that the Bayley III can be used to assess developmental outcome in inner city southern African children. Cognitive, language, and motor scores were within the normal range, but below the mean of 100, possibly due to social and cultural factors related to the study population. There was significant variation in Bayley III scores performed before and after one year of age. A control group of normal children from the same population and age should therefore be used when reporting developmental outcome. A cut-off of 70 was appropriate for defining developmental delay in the present study; children with a score below 85 can be reported to be “at risk.”

## Figures and Tables

**Table 1:** Sociodemographic factors of children undergoing a Bayley III assessment.

Variable	*n* (%)
*Mother's education*	65
Primary school	15 (23)
Grade 10	27 (41.5)
Matric	17 (26.1)
Tertiary education	6 (9.2)
*Mother's employment*	
Unemployed	42 (64.6)
Part time employment	13 (20.0)
Full time employment	9 (13.8)
*Housing*	
House	18 (27.6)
Apartment	35 (53.8)
Shack	12 (18.5)
*Household amenities*	
Electricity	61 (93.8)
Running water	60 (92.3)
Sanitation	59 (90.7)
Family car	16 (24.6)

**Table 2:** Bayley III assessments at different ages in the same children (*n* = 47).

Variable	First assessment		Second assessment		*p* value
	Mean	95% CI	Mean	95% CI	
Adjusted age	10.9	10.1–10.7	19.9	19.1–20.7	<0.001
Cognitive	92.8	89.5–96.1	93.2	89.3–97.1	0.874
Language	103.0	99.7–106.2	94.6	91.7–97.5	0.001
Motor	95.2	92.2–98.3	100.1	97.9–102.4	0.004

**Table 3:** Composite scores below 85 in the 47 paired Bayley III assessments.

Composite score below 85	First assessment *n* (%)	Second assessment *n* (%)	*p* value
Cognitive	8 (17.0)	11 (23.4)	0.441
Language	3 (6.3)	6 (12.7)	0.293
Motor	3 (6.3)	1 (2.1)	0.307

## Additional Points

*Limitations of the Study*. There was a drop-out rate of 16.8% in the current study, despite sending appointment reminders and refunding transport costs. Many of the children were from other provinces or countries and the children are sent home when they reach one year of age. Once parents return to work, they are more reluctant to bring well children to the hospital for follow-up. The sample size was relatively small, so the effect of sociodemographic factors on childhood development could not be fully evaluated.
